# Motor Competence in Greek Adolescents Using Movement Assessment Battery for Children—2nd Edition: Associations with Individual and Environmental Factors

**DOI:** 10.3390/children13091260

**Published:** 2026-09-16

**Authors:** Ermioni Katartzi, Maria Kontou

**Affiliations:** Department of Physical Education and Sport Science at Serres, Faculty of Physical Education and Sport Science, Aristotle University of Thessaloniki, Agios Ioannis, 62100 Serres, Greece; marikont@phed-sr.auth.gr

**Keywords:** motor difficulties, gender, grade, body mass index, handedness, physical activity, obesity

## Abstract

**Highlights:**

**What are the main findings?**

- Exploratory findings indicated a relatively low prevalence of motor difficulties among Greek adolescents (1.5% definite, 3.6% at risk), with preliminary trends suggesting potential gender-based variations in specific skill areas.
- Preliminary trends suggest that extracurricular organized physical activity might serve as a potential compensatory asset for adolescents experiencing motor difficulties.

**What are the implications of the main findings?**

- Exploratory observations suggest that future intervention frameworks could consider potential gender-specific trends, with a tentative focus on balance and manual dexterity for boys and ball skills (object control) for girls.
- Hypothesis-generating observations suggest that extracurricular organized physical activity programs could potentially serve as a supportive framework for adolescents presenting with lower motor competence.

**Abstract:**

Background/Objectives: The primary objective of the present study was to assess motor competence and identify motor difficulties among Greek adolescents using the Movement Assessment Battery for Children—2nd Edition (MABC-2). The secondary aim was to evaluate associations and differences across gender, school grade (age), BMI, hand dominance, extracurricular organized PA participation, and school type in relation to motor competence. Methods: The MABC-2 was utilized primarily as an exploratory screening instrument to identify potential motor difficulties within the sampled adolescent cohort (***N*** = 742; *M*_age_ = 13.98 yrs.), rather than as a definitive clinical diagnostic tool for Developmental Coordination Disorder. Results: This study identified a low prevalence of motor difficulties among the sampled adolescents (1.5% definite, 3.6% at risk). Preliminary trends suggested a 1.5:1 boy-to-girl motor difficulty ratio. Within exploratory analyses, “at-risk” boys scored lower in balance and manual dexterity, while girls showed potential challenges in ball skills. Tentative subgroup analyses indicated that physical activity participants within these cohorts scored higher in balance and ball skills, though small sample sizes warrant caution. Other demographic or school environmental factors—specifically academic grades, body mass index, handedness, and school types—showed no clear associations with motor competence. Conclusions: This study identified a low prevalence of motor difficulties among Greek adolescents, with preliminary trends suggesting that structured extracurricular activity may support motor development. The findings indicate potential gender variations in specific motor domains and highlight the need for longitudinal research to confirm these observations.

## 1. Introduction

Developmental Coordination Disorder (DCD) is a discrete neurodevelopmental disorder characterized by motor difficulties that disrupt daily tasks, academic performance, recreational activities, and sports. Crucially, these difficulties occur in the absence of any neurological or pathological etiology. According to the Diagnostic and Statistical Manual of Mental Disorders (DSM-5), DCD diagnosis relies on four distinct criteria: “(A) acquisition and execution of coordinated motor skills are substantially below that expected given the individual’s chronological age and opportunity for skill learning and use. Difficulties are manifested as clumsiness as well as slowness and inaccuracy of performance of motor skills; (B) motor skills difficulties in Criterion A significantly and persistently interfere with activities of daily living appropriate to chronological age (e.g., self-care and self-maintenance) and impact academic/school productivity, prevocational and vocational activities, leisure, and play; (C) onset of symptoms is in the early developmental period; and (D) motor skill difficulties are not better explained by intellectual disability or visual impairment and are not attributable to a neurological condition affecting movement” [1]. While DCD diagnosis is a multidimensional process, utilizing the Movement Assessment Battery for Children—Second Edition (MABC-2) alongside the DSM-5 criteria allows for accurate and detailed identification in adolescents. This comprehensive approach enables the design of beneficial, targeted interventions. However, individual assessments must account for confounding factors such as age and environment to ensure personalized support [1,2,3,4,5,6]. Despite its high prevalence, DCD remains one of the most underrecognized disorders within medical and educational systems, making timely diagnosis crucial [7,8]. In the present study, because the full DSM-5 clinical diagnostic criteria were not explicitly evaluated, a MABC-2 total score at or below the 15th percentile was categorized as “motor difficulties” rather than a clinical DCD diagnosis [3,4,5,6]. Consequently, the terms “motor difficulties” and “low motor competence” are used interchangeably throughout this paper.

Although the most widely accepted prevalence estimate of DCD in children is 5% to 6%, cumulative evidence suggests this figure may range from 2% to 20% [1,9,10]. Recent school-based studies in children and adolescents across various cultures have yielded ambiguous results, reporting prevalence rates either slightly higher [1,9,11] or lower than historical estimates [12]. These discrepancies are largely driven by the varying strictness of the selection criteria applied to identify motor difficulties, which heavily impacts epidemiological data [9]. Furthermore, evidence indicates that without timely intervention, these motor challenges persist into adolescence and adulthood for 50% to 70% of affected individuals, continuously impacting multiple aspects of their daily lives [1]. As children facing motor difficulties grow, their overall motor performance advances. However, their skill development trajectory is rarely linear or sequential, a phenomenon often attributed to reduced participation in physical activity (PA) [13]. Furthermore, balance competence—a critical factor for precise and coordinated motor development—is significantly impaired in children and adolescents with DCD. This deficit further diminishes their performance in daily activities and exacerbates their avoidance of PA [14,15,16].

Epidemiological studies on the gender distribution of motor difficulties indicate that the condition is more frequently diagnosed in boys than in girls, with the male-to-female ratio ranging between 2:1 and 7:1 [1,9]. Conversely, a study from India involving school-aged children (6–15 years old) reported a boy-to-girl ratio of 1:2, indicating a higher prevalence among girls [12]. These gender differences could be attributed to variations in foundational neurobiological mechanisms between males and females [17]. Additionally, methodological discrepancies, varying diagnostic criteria, and cultural factors—such as differing opportunities for learning and practicing motor skills—may significantly influence reported gender ratios in motor difficulty prevalence [9,18].

In recent years, overweight and obesity have been increasingly associated with developmental disorders, and, more specifically, with DCD [19,20]. Excessive body weight during childhood and adolescence elevates the risk of adult obesity and its related chronic diseases [21]. Research indicates that motor difficulties detrimentally impact both psychological and physical well-being, including fitness levels. This decline is often mediated by lower rates of engagement in PA and increased sedentary behavior [22,23,24]. Consequently, children with motor difficulties frequently display a higher body mass index (BMI) alongside deficits in cardiorespiratory endurance, strength, flexibility, and coordination. These limitations predispose them to a significantly higher risk of chronic, obesity-related diseases compared to their typically developing peers [6,11,25,26,27,28,29,30,31,32,33,34], a vulnerability that intensifies with age [20]. Furthermore, adolescents with motor difficulties often fail to meet general PA recommendations, largely due to a lack of enjoyment in organized sports [33,35]. Conversely, active PA participation has been shown to positively influence motor skill performance, daily functioning, and overall well-being [36]. The activity deficit hypothesis and the resulting negative cycle of inactivity further justify the assumption that motor difficulties suppress motivation for PA, thereby reinforcing low motor competence [35,37,38]. Ultimately, these early challenges can predict reduced PA and enhanced sedentarism in adulthood, underscoring the urgent need for timely assessment and identification during childhood and adolescence [24].

Inconsistent handedness and left-handedness have been frequently associated with DCD [39,40]. The general consensus suggests that a higher incidence of left-handedness occurs among individuals with DCD and other developmental disabilities than in the general population [40]. This hypothesis is supported by several studies confirming an elevated prevalence of left, unstable, and mixed handedness in school-aged children with DCD [39,41], although earlier research has reported ambiguous results [42].

Given that DCD remains an underdiagnosed and poorly funded motor learning impairment, the educational environment—particularly physical education (PE) classes—offers an ideal venue for enhancing motor proficiency and fostering active lifestyle participation among adolescents [7,43,44]. In this context, previous research indicates that school environments, such as those in private institutions, can positively affect motor development in early childhood through distinct structural organizations and specialized curricula. Moreover, preschoolers in public schools are more likely to present severe motor difficulties and lower scores across all MABC-2 domains compared to those in private schools [45,46]. In Greece, secondary school students spend approximately six hours per day at school. Public and private secondary institutions follow the same core curriculum policy, offering identical academic lessons and PE classes twice a week. However, private schools operate independently and can offer extracurricular sports, cultural, and artistic activities. While public schools are funded exclusively by the state—relying entirely on educational administration for material and human resources—private schools are funded through tuition fees paid by families. Due to market competition, private schools often feature superior facilities and broader sports participation opportunities to attract enrollment [47]. In contrast, public music schools in Greece follow a specialized curriculum focused primarily on music classes, though they maintain standard PE classes twice a week [48]. Evidence indicates that even simple music-listening activities provide cognitive benefits for both typically and atypically developing brains, highlighting musical practice as a potent tool for neuroeducation. Furthermore, music training stands out among various extracurricular activities (including sports, visual arts, and drama) for its prominent role in optimizing children’s executive functions. This optimization stems directly from the complex integration of sensory processing and motor skill performance required during training [49,50]. Nevertheless, individuals experiencing motor difficulties have to invest substantial additional cognitive resources to acquire and execute daily motor skills, as well as to manage their personal schedules, time, and materials [51]. Crucially, to the best of our knowledge, no prior research has examined the association between the prevalence of adolescent motor difficulties and different school types.

According to Newell’s Model of Constraints, individual and environmental factors, alongside their interactions, play a critical role in motor development during childhood and adolescence [1,2,52]. Furthermore, the impact of motor difficulties on an individual is heavily mediated by their unique personal traits and the strength of their social support system [9]. Therefore, both individual and environmental factors can alter the typical course of motor development and must be carefully evaluated during the identification of motor difficulties. Understanding how variables such as age (or educational level), sex, elevated body mass, hand dominance, PA engagement, and school type relate to adolescent motor difficulties can inform the design of tailored interventions. This targeted approach is vital for enhancing motor competence, especially since educational institutions serve as pivotal environments for supporting these students [7,43].

We initially hypothesized that the prevalence and gender ratios of adolescent motor difficulties, as identified by the MABC-2, would align with the existing literature. Establishing this baseline hypothesis was essential, given that these epidemiological estimates have not yet been formally validated in adolescents within the Greek context. Secondly, we hypothesized that gender would be significantly associated with motor competence classification across the three MABC-2 zones, and that adolescents with motor difficulties would exhibit distinct MABC-2 scores based on their gender. Similarly, the third hypothesis posited that PA participation would be associated with motor competence zones, and that MABC-2 scores for adolescents with motor difficulties would differ according to their level of PA engagement. Finally, we hypothesized that body mass index (BMI), hand dominance, school grade (age), and school type would all be significantly associated with the three-zone motor competence classification.

Therefore, the main purpose of the present study was firstly to assess motor competence and identify motor difficulties among Greek adolescents using the MABC-2 and secondly to evaluate associations and differences across gender, school grade (age), BMI, hand dominance, extracurricular organized PA participation, and school type in relation to motor competence.

## 2. Materials and Methods

### 2.1. Participants

A one-stage cluster sampling method was employed for standard public-school selection. Out of the six available standard public schools (clusters), three were randomly selected, and all eligible adolescents within these schools were invited to participate. In addition, a purposive cluster sampling approach was adopted for public music and the standard private schools. Both specific schools were selected to meet the study’s inclusion criteria, and a total census of all eligible students within each school was conducted. Thus, the sample comprised 742 adolescents (367 males, 49.5%; 375 females, 50.5%) recruited from five distinct educational institutions in an urban, middle-class region of Northern Greece. Specifically, these included three standard public secondary schools, one public music school, and one standard private secondary school. Consequently, the findings are uniquely representative of similar socioeconomic urban settings. In total, 47 participants (6.3%) attended 1st grade, 413 (55.7%) 2nd grade and 282 (38.0%) 3rd grade, with a mean age of 13.98 yrs, (*SD* = 0.74), mean body height of 165.93 cm (*SD* = 8.70) and mean body weight of 56.79 kg (*SD* = 10.91). In total, 292 (39.4%) of them participated in extracurricular organized PA, mostly in team sports such as soccer, basketball, volleyball, handball and water polo (56.7%), while 35% of them participated in individual sports such as swimming, track and field, martial arts and dancing activities, and 8.3% in dual sports like tennis and badminton. In total, 292 reported a mean weekly frequency of participation in extracurricular organized sports of 3.30 times/week (*SD* = 0.88), with a mean of 72.55 min per training session (*SD* = 22.64). In addition, 90.4% of them reported to be right-handed. Finally, according to BMI classification (Table 1), 2.5% were classified as underweight, 78% as having healthy weight, while 15.3% and 4.2% as overweight and obese, respectively.

### 2.2. Materials

Movement Assessment Battery for Children—2nd Edition (MABC-2) age band three (11–16 years and 11 months) was used for adolescents’ assessment. This motor test is designed to identify motor difficulties and assess motor competence in children and adolescents, while providing information and quality observations regarding motor development and effectiveness of motor intervention programs [4]. The 3rd age band of MABC-2 consists of eight test items divided into three categories: manual dexterity (turning pegs, triangle with nuts and bolts, drawing trail 3), ball skills/aiming and catching (catching with one hand, throwing at wall target) and static and dynamic balance (two board balance, walking toe-to-heel backwards, zigzag hopping). A practice attempt and two official trials are given with the best to be evaluated. If the individual cannot complete the attempt properly it is classified as failed (F), if they refuse to perform it, as a refusal (R) and if they have inappropriate behavior during execution, as inappropriate (I).

According to the manual’s norms, raw scores derived from each test item are converted into age-adjusted item standard scores (SSs) and component standard scores (CSSs), across the three motor domains and the total motor test score. The total standard score can be transformed into an age-adjusted percentile rank, and this ranking is applied in the three motor domains and the total motor test score. The MABC-2 manual, according to a “traffic light system”, classifies individuals who score at or below the 5th percentile (≤5th) (red zone—total test score up to and including 56) as having a significant motor difficulty, while those who score between the 6th and 15th percentile (amber zone—between 57 and 67 inclusive for total test score) as being “at risk” of having a motor difficulty and requiring monitoring. Adolescents who score above the 15th percentile (>15th) (green zone—any total test score above 67) do not have a motor difficulty [4]. Clinically, a cut-off score of ≤15th percentile to diagnose DCD is recommended, while a stricter cut-off score of ≤5th percentile is recommended for children aged 3–5 years [10]. However, the selection of both the 5th and 15th percentiles in the present study directly aligns with the standardized scoring protocol of the Movement ABC-2. Rather than using a rigid binary classification (typical vs. atypical), the test utilizes a ‘traffic light system’ designed to capture a gradient of motor impairment, which this study adopts to ensure methodological fidelity. BMI was calculated and converted to sex- and age-specific percentiles using the CDC growth charts for children and adolescents 2 through 19 years old [53]. According to CDC criteria, weight status classification is presented in Table 1.

### 2.3. Procedure and Design

The study was approved by the institutional research ethics committee (50134/2024) before the experiment started and was conducted in accordance with the principles set forth in the Helsinki Declaration. All parents or legal guardians of adolescents were informed about testing procedures, and corresponding written consent was obtained prior to participation. Also, the adolescents’ consent was obtained for their involvement and access to relevant information. Adolescents with a documented history of intellectual, physical, or emotional disabilities, as well as those with special educational needs, were excluded from the study. This criterion was essential because motor difficulties due to DCD is a discrete developmental disorder, and incorporating individuals with other co-occurring disabilities would compromise the validity of the results [1]. Prior to data collection, permission to conduct the study was granted from the school headmasters. The purpose of the study was explained to the participants; they were informed that their participation was voluntary and that their examination would remain confidential. None of the participants denied participating in the data collection. A coding system was used to secure anonymity. A screening procedure using MABC-2 was performed to identify adolescents with motor difficulties. At this stage, adolescents were assessed individually in a specially equipped, quiet area and we required about 20 min to complete the assessment.

### 2.4. Data Analysis

Due to the exploratory nature of this study and the wide range of variables evaluated, multiple statistical tests were performed across different motor domains and subgroups. Readers should note that conducting multiple simultaneous comparisons may inflate the family wise Type I error rate. Consequently, these analyses are intended primarily for hypothesis generation and trend identification rather than definitive hypothesis testing, and the corresponding p-values should be interpreted with appropriate caution. Categorical variables were presented as absolute frequencies (*n*) and relative frequencies (%), while quantitative variables were presented either as means and standard deviations (if they followed the normal distribution) or as medians and amplitudes (if they did not follow the normal distribution). The Kolmogorov–Smirnov and Shapiro–Wilk tests, as well as Q-Q Plots, were used to check the normal distribution of the quantitative variables. The chi-square test of independence and the Fisher exact test were used to investigate the relationship between two categorical variables. Between a quantitative variable (following the normal distribution) and a categorical one (with two categories), the independent-samples *t*-test was used. For non-normally distributed data across three distinct categories, a non-parametric Kruskal–Wallis test was applied. Effect sizes for independent-samples *t*-tests were calculated using *Hedges’ g* estimator [54]. The magnitude of the effect sizes was interpreted based on Cohen’s benchmarks [55], where values <0.2, 0.2–0.5, 0.5–0.8, and >0.8 were classified as negligible, small, medium, and large, respectively. Moreover, the eta-squared (*η*^2^) effect size based on the H-statistic was calculated for the Kruskal–Wallis test to evaluate the strength of the results. *η*^2^ values < 0.01, 0.01–0.06, 0.06–0.14 and >0.14 denote negligible, small, medium and large effects, respectively [55,56]. Statistical significance was set at a two-tailed alpha level of 0.05. The statistical processing and analysis of the survey data were performed using the statistical package IBM SPSS Statistics, version 29.0 (IBM Corp., Armonk, NY, USA).

## 3. Results

### 3.1. Anthropometric Characteristics by MABC-2 Zones of Motor Difficulties

Anthropometric characteristics across the three MABC-2 motor competence zones are presented in Table 2. Because the assumption of normality for the total sample (*N* = 742) was violated for all anthropometric variables (Kolmogorov–Smirnov and Shapiro–Wilk tests, *p* < 0.05), the non-parametric Kruskal–Wallis test was used to examine differences among the zones, with results showing no significant differences in mean ranks for age, body height, body weight, and BMI (*p* > 0.05). To determine the magnitude of these differences, the eta-squared (*η*^2^) effect size based on the H-statistic was calculated, utilizing medians and mean ranks for all inferential statistical analyses [57] (Table 2).

### 3.2. Prevalence of Motor Difficulties According to MABC-2 Scores in Adolescents: Individual Factors

Based on the MABC-2 “traffic light system,” 94.9% of participants showed no motor difficulties, with 3.6% in the “at-risk” zone and 1.5% in the “definite motor difficulties” zone, revealing no significant gender differences. A chi-square test of independence (in the Fisher–Freeman–Halton exact test, 16.66% of the cells had an expected frequency of less than five) revealed no statistically significant association between gender and overall MABC-2 classification (*χ*^2^ = 1.23, *p* = 0.512). Performance varied by domain, with the aiming/catching subscale showing the highest rate of definite difficulties (6.1%), while static/dynamic balance showed the highest green zone classification (95.8%). The results are depicted in Table 3.

Exploratory independent-samples *t*-tests (equal variances assumed—Levene’s test of equality of variances; *p* > 0.05) suggested potential gender differences in the aiming/catching domain for both the “at-risk” and “definite motor difficulties” cohorts (Kolmogorov–Smirnov and Shapiro–Wilk test, *p* > 0.05 for these zones), with boys scoring higher than girls. No statistically significant gender differences were observed for other domains, and descriptive trends in manual dexterity did not reach significance; effect sizes were calculated using *Hedges’ g*. These findings are preliminary and hypothesis-generating rather than definitive (Table 4).

A Pearson chi-square (*χ*^2^) test of independence, utilizing the Fisher–Freeman–Halton exact test because 50% of cells had expected frequencies below five, evaluated the association between MABC-2 motor competence zones and BMI classifications. The “Severe Obesity” category was omitted from the omnibus analysis due to zero cell frequencies across all strata. The analysis indicated no statistically significant relationship between motor competence zones and weight status (*χ*^2^ = 7.54, *df* = 6, *N* = 742, *p* = 0.274). Descriptively, the rate of overweight was 15.1% in the green zone, 22.2% in the amber zone, and 27.3% in the red zone, while obesity rates stood at 4.0%, 3.7%, and 18.2% across the respective zones (Table 5).

A Pearson chi-square test, using the Fisher–Freeman–Halton exact test, indicated no statistically significant relationship between hand dominance and MABC-2 motor competence zones (*χ*^2^ = 3.69, *df* = 2, *N* = 742, *p* = 0.158). While 7.0% of left-handed participants fell into the amber zone compared to 3.3% of right-handed peers, zero left-handed participants were in the red zone (Table 6).

A Pearson chi-square (*χ*^2^) test of independence, utilizing the Fisher–Freeman–Halton exact test due to 30% of cells having expected frequencies below five, evaluated whether the distribution of MABC-2 motor competence zones differed across secondary school grade levels. The analysis indicated no statistically significant relationship between academic grade levels and motor competence zones (*χ*^2^ = 5.31, *df* = 4; *N* = 742, *p* = 0.257). Green zone classifications remained stable between 93.6% and 95.0% across all grade cohorts. Descriptively, the prevalence of definite motor difficulties (red zone) was 1.4% in the first grade, 0.7% in the second grade, and 2.5% in the third grade (Table 7).

### 3.3. Prevalence of Motor Difficulties According to MABC-2 Scores in Adolescents: Environmental Factors

A Pearson chi-square (*χ*^2^) test of independence revealed no significant relationship between Movement ABC-2 motor competence zones and participation in extracurricular organized physical activity (*χ*^2^ = 0.47, *df* = 2, *N* = 742, *p* = 0.789). Active enrollment rates were similar across groups, with 39.6% in the green zone, 33.3% in the amber zone, and 36.4% in the red zone. The analysis suggests that motor competence levels do not significantly influence an adolescent’s enrollment in extracurricular physical activities within this sample (Table 8).

Exploratory independent-samples *t*-tests (for equal variances assumed—Levene’s test of equality of variances *p* > 0.05) evaluated the potential effects of extracurricular organized PA participation on specific MABC-2 motor domains within the amber (*N* = 27) and red (*N* = 11) zones (Kolmogorov–Smirnov and Shapiro–Wilk tests, *p* > 0.05 for the above zones), with effect sizes calculated using *Hedges’ g*. Within the amber zone, trends suggested that non-participants performed better in manual dexterity, whereas PA participants showed higher static/dynamic balance scores. No significant differences were observed for aiming/catching or total MABC-2 scores, nor for most domains in the red zone, with the exception of aiming/catching. Given the exploratory nature of these analyses, these preliminary observations serve primarily as hypothesis-generating trends rather than definitive conclusions (Table 9).

A Pearson chi-square (*χ*^2^) test, using the Fisher–Freeman–Halton exact test (33.33% of the cells had an expected frequency of less than five), showed no significant association between school type (standard public, public music, standard private) and Movement ABC-2 motor competence zones (*χ*^2^ = 7.61, *df* = 4, *N* = 742, *p* = 0.107). While 100% of public music school students were in the typical (green) zone compared to 92.5% and 92.1% in standard public and private schools, respectively, these differences were not statistically significant (Table 10).

## 4. Discussion

The aims of the present study were to assess motor competence and identify motor difficulties among Greek adolescents using the MABC-2 and to evaluate associations and differences across gender, school grade (age), BMI, hand dominance, extracurricular organized PA participation, and school type in relation to motor competence.

According to the first hypothesis regarding the identification of motor difficulties, the results indicated that 1.5% of adolescents revealed definite motor difficulties, whereas 3.6% were classified as “at risk” of having a motor difficulty, as tested by the MABC-2. The highest percentage of adolescents with definite motor difficulties presented low motor competence mainly in ball skills, while in “at risk” adolescents, the highest percentage was in manual dexterity. These results are in line with the general estimations of 2% to 20% for motor difficulties, revealing a relatively low prevalence that is close to the international standards reported in children and adolescents [58]. Previous research among younger school age children, in Greece, indicated that the prevalence of definite motor difficulties was 1.6%, whereas about 10% was estimated as being “at risk” [59]. Moreover, the prevalence of motor difficulties in adolescents in the present study is in line with results in Europe, as the motor difficulty estimate was about 2%, lower than in North America (6%), Brazil (11.6%) and Asia (4%), whereas it is higher than estimates in South India (0.8%) [12,58,60]. Discrepancies in motor difficulty prevalence rates may stem from variations in assessment instruments, as well as individual (e.g., age, gender) and environmental influences (e.g., school type, sports involvement). Consequently, accounting for these variables in motor difficulty prevalence epidemiological research is essential for a more precise interpretation [9]. There is notable heterogeneity in the international literature, with some epidemiological studies adopting an exclusive ≤5th percentile threshold for high specificity, and others employing the broader ≤15th percentile threshold for higher sensitivity, when using MABC-2. By tracking both cut-off points, this study ensures maximum comparability with both conservative clinical trials and wider population-based screenings [10]. However, the identification of motor difficulties is a multi-dimensional procedure and should consider factors, such as age and environment, for specific and personalized interventions designs [1,2].

Secondly, in alignment with the second hypothesis, findings indicated that boys exhibited a marginally greater rate of motor difficulties than girls. Specifically, males’ lower competence was predominantly observed in balance and manual dexterity, whereas females’ lower competence centered on object control (ball skills). Nonetheless, no association was established between gender and the three zones of motor competence. These outcomes corroborate the current literature, which consistently demonstrates a higher prevalence of motor impairments among males than females, with reported male-to-female ratios spanning from 2:1 to 7:1 [1,9,58], though research from India counterintuitively demonstrated a higher prevalence among girls than boys [12]. Moreover, differences in underlying neurological systems (brain size and volume, density of gray matter, cerebral blood flow and thickness of the cortical areas) between boys and girls may also explain this unclear relationship [17,58,61]. Regarding motor domains, the results of the present study showed that motor difficulties in balance (static and dynamic) were more prevalent among boys over girls, in agreement with other studies which indicated that boys may experience greater postural control challenges [62], as it is a motor domain that individuals with motor difficulties experience low motor competence [16]. On the other hand, girls with low motor competence showed difficulties with ball skills as boys were found to be more proficient in object control skills, such as throwing, catching, and kicking [63].

However, subgroup analyses for the amber and red zones are considered strictly exploratory and warrant cautious interpretation due to limited sample sizes within these cohorts. These findings should be viewed as hypothesis-generating trends rather than definitive results. These insights suggest that gender-related variations in the prevalence of motor difficulties among children and youth may diverge across cohorts due to methodological differences, diagnostic thresholds, or cultural influences. Consequently, these exploratory observations highlight the potential value of unified assessment instruments and longitudinal research frameworks to clarify these trends [9,18].

Consistent with the third hypothesis, the findings indicated that participation in extracurricular organized PA was not significantly associated with motor competence classification across the three MABC-2 zones. Moreover, exploratory subgroup analyses suggested that adolescents within both the “at-risk” and “definite motor difficulties” cohorts who engaged in organized extracurricular PA tended to exhibit higher scores in static and dynamic balance, as well as aiming and catching (ball skills), compared to their non-participating counterparts. The existing literature underscores that individuals with motor difficulties frequently avoid engaging in physical and recreational activities, an observation that aligns with the activity deficit hypothesis and the conceptual framework of a negative cycle of inactivity [25,34,35,38]. Furthermore, recent evidence indicates that youth with lower motor competence may often struggle to meet standard PA recommendations and frequently report less enjoyment in structured sports [33,64]. Due to perceived low social status among peers, some individuals with motor difficulties may avoid PA, potentially leading to a further decline in their physical fitness and motor skills. This pattern can create an ongoing barrier to PA participation, with research suggesting that physical withdrawal may undermine motor development, daily task performance, and psychological well-being [36,37]. Such reductions in PA and heightened sedentary habits could persist into adulthood, suggesting that motor challenges and their secondary impacts may extend to later stages of life. While the present study did not observe a significant statistical association between overall PA involvement and motor difficulty classification, preliminary trends within the subgroup analyses suggested that adolescents with motor challenges who participated in structured PA tended to exhibit higher scores in balance and object control skills. This appears consistent with the framework where targeted PA potentially pairs with adaptive strategies in youth with motor limitations, particularly since balance deficits may be closely associated with restrictions in daily functioning [14,15,16]. Consequently, these observations suggest the potential value of evaluating and identifying both motor difficulties and PA patterns during childhood and adolescence. Such an approach could inform the timely development of targeted, activity-specific interventions [24,64]. Educational settings may serve as a supportive channel for delivering varied PE programs designed to foster motor competence in both typically developing youth and those experiencing low motor competence challenges [25,65]. The emerging patterns within the amber and red zones should be interpreted cautiously as preliminary trends, given that the limited cell frequencies in these specific cohorts constrain the broader generalization of the findings. Moreover, when interpreting these trends, it is essential to acknowledge that our evaluation of PA was limited to a basic, dichotomous measure of participation. Consequently, these exploratory findings should not be overextended to imply a direct or definitive impact of physical activity on overall motor competence. Because our metric did not capture nuanced dimensions such as the precise volume, frequency, intensity, or qualitative nature of the physical activities, these observations serve strictly as preliminary indicators. They suggest potential avenues for future research rather than establishing a conclusive causal relationship between extracurricular PA and motor proficiency.

In exploring the final hypothesis, the analyses indicated that academic grade (age), BMI, handedness, and school type were not significantly associated with motor competence classification across the three MABC-2 zones. With respect to age, the descriptive data suggested that motor difficulty classification rates remained relatively constant across the three secondary school grades within this sample. Although motor competence typically advances with age, the literature suggests that this developmental trajectory can be disrupted in individuals with motor difficulties, potentially linked to a decreased engagement in PA [13,63]. The literature suggests that balance deficits in children and adolescents with motor difficulties may potentially affect their performance in daily living activities. Consequently, these challenges could restrict their participation in PA, which might further influence their overall motor competence. Future studies are warranted to investigate the specific factors that may underlie these divergent motor development trajectories in adolescents experiencing motor difficulties [14,15,16]. This is particularly crucial given that adolescence is a highly dynamic phase of physical, psychosocial, and individual growth, during which the pubertal growth spurt can induce transient declines in motor competence—a phenomenon widely recognized as “adolescent awkwardness” [3,63]. Age may be viewed as a factor that interacts with the biological and neurological maturation of the individual, as well as their surrounding environment. Within this framework, potential variations in motor development across age groups could be linked to the continuous interaction between maturation and environmental factors [66]. Given that age can be an important consideration in motor competence profiles during adolescence, it may provide valuable context during screening procedures aimed at identifying specific subgroups with lower motor competence [63].

Regarding BMI, the descriptive findings of the present study suggested that motor difficulty classification rates appeared relatively uniform across BMI categories within this sample. This observation diverges from trends in some established literature, which frequently reports positive associations between adolescent motor skills and PA levels, alongside an inverse relationship with body weight. While the literature often points to a potential co-occurrence of motor difficulties with overweight and obesity—noting that this relationship may strengthen with age [3,20] and could be linked to reduced PA participation and increased sedentary behaviors [22,23,24]—the exploratory nature of the current analyses warrants a cautious interpretation of these specific variables. Since adolescent motor challenges may potentially extend into adulthood alongside elevated body weight and secondary health risks, evaluating weight status during the exploratory screening of motor difficulties could offer valuable context. Integrating considerations of biological maturation might also support the overall interpretation of these profiles [24]. Consequently, future studies utilizing a longitudinal research design could help clarify the directionality of the relationships among these variables.

When accounting for hand preference, the descriptive data from the current study suggested that lateral preference was not significantly associated with motor difficulty classification rates within this sample. This observation diverges from some previous research, which has highlighted that motor challenges can be associated with left-handedness or inconsistent lateral preference [40]. While this phenomenon is noted among some younger school-aged cohorts experiencing lower motor competence, the existing literature occasionally yields contradictory findings [39,41,67]. Although these exploratory observations did not confirm our initial hypothesis, they appear partly consistent with certain published trends [42]. However, these results require cautious interpretation given the limited sample size within the specific motor difficulty subgroups. Because the potential relationships between lateral preference and motor difficulties require further clarification, future research should continue to examine the nuances associated with hand dominance in this population to help inform future educational and supportive frameworks [40].

Exploratory analyses did not indicate a significant association between motor difficulty prevalence and school type, contrasting with the previous literature suggesting private schools offer superior physical development [44,45,46]. This similarity is likely due to the unified national curriculum in Greek secondary schools, unlike the more flexible frameworks found in preschool settings [47]. The absence of low motor competence among students in music schools is a descriptive and highly tentative finding, rather than evidence of a protective curriculum effect, reflecting potential self-selection and unmeasured confounders [48]. Key factors likely include pre-existing motor profiles, higher socioeconomic status, parental support, or specific cognitive traits among the sampled students [68]. Future studies should treat these patterns as hypothesis-generating, demanding multi-variable analysis to understand how varied school environments impact physical growth, acknowledging that specific school contexts significantly influence motor development trajectories [52].

## 5. Conclusions

This study identified a relatively low prevalence of lower motor competence among the sampled adolescents (1.5% definite and 3.6% at risk), aligning with several European and international benchmarks. While boys in this sample exhibited a marginally higher rate of motor challenges than girls, exploratory trends suggested that specific areas of difficulty may diverge by gender. Within these tentative observations, boys classified with lower motor competence tended to present lower scores primarily in balance and manual dexterity, whereas girls in the same cohort showed potential challenges in ball skills (object control). Moreover, this study highlights the potential utility of a nuanced, two-tiered assessment approach when evaluating pediatric motor proficiency. By employing both the 5th and 15th percentile cut-off points of the Movement ABC-2, this exploratory framework allowed for a distinction between children with definite motor impairments and those exhibiting borderline, “at-risk” profiles. This dual-threshold classification suggests that motor difficulties may exist on a continuum rather than a simple binary scale. Capturing both cohorts could offer valuable insights into public health and educational planning, helping to identify children who may benefit from immediate therapeutic and/or educational intervention alongside those who might require preventive monitoring to mitigate long-term functional and socio-emotional challenges. In addition, while overall motor competence classification across the three MABC-2 zones does not appear to be statistically linked to extracurricular organized PA, exploratory subgroup analyses suggested potential trends toward a compensatory effect. Adolescents in both the “at-risk” and “definite motor difficulties” cohorts who engaged in organized PA tended to exhibit higher scores in balance and ball skills compared to their non-participating peers, though these observations require caution due to limited sample sizes. Furthermore, individual and school-related environmental determinants—specifically academic grade, BMI, handedness, and school types—showed no significant association with the overall motor difficulty classification. Notably, the complete absence of low motor competence within the music school cohort warrants further investigation, as this outcome may be linked to unmeasured confounding variables. Although the present study was conducted within secondary schools in a middle-class urban area of Northern Greece, the rigorous sampling methodology employed allows the findings to be considered representative of comparable socioeconomic urban settings and not fully representative of Greek adolescents nationwide. A notable limitation of the present study is that the full clinical diagnostic criteria outlined in the DSM-5—such as a detailed developmental history, medical examinations to exclude neurological or sensory conditions, and verification of functional impact across environments—were not explicitly evaluated. Consequently, the prevalence rates and classifications reported herein strictly reflect performance on the MABC-2 screening tool and must be understood as indices of ‘low motor competence’ or ‘motor difficulties’ rather than a clinical diagnosis of DCD. Readers should exercise caution when comparing these findings with data derived from clinically confirmed DCD populations. Furthermore, these exploratory findings are constrained by low statistical power resulting from small subgroup cell sizes, which may compromise the stability of these specific comparisons. The study is also limited by a cross-sectional design that precludes longitudinal analysis of motor difficulties, and a subjective, simple, binary variable (participation vs. non-participation) approach to evaluate extracurricular organized PA. This dichotomous classification is insufficient for capturing the complex, multi-dimensional nature of PA, as it fails to account for critical parameters such as the frequency, duration, intensity, or the specific qualitative modalities of the activities performed. Consequently, this broad metric may obscure important variations in how physical activity interacts with motor proficiency profiles. To address these limitations, future research could consider employing longitudinal frameworks to better track the potential temporal stability and developmental trajectories of motor difficulties into adulthood. Future investigations could benefit from utilizing larger, more diverse samples to help enhance statistical power and support more robust subgroup comparisons. Methodologically, incorporating comprehensive diagnostic protocols that address all DSM-5 criteria—such as combining motor competence testing with developmental histories, daily functioning measures, parent or teacher reports, and clinical interviews—could provide valuable support for confirming a formal DCD diagnosis. Finally, to overcome the substantial methodological gap regarding PA assessment, future investigations should employ comprehensive, multi-item questionnaires alongside objective metrics—such as accelerometers and heart rate monitors—to provide a highly nuanced, quantitative, and reliable representation of adolescents’ physical activity levels. Consequently, these limitations warrant a cautious interpretation of the observed trends, as the data cannot verify long-term motor difficulty patterns or provide a highly nuanced representation of PA levels. Ultimately, this study identified a relatively low prevalence of motor difficulties among Greek adolescents, pointing to potential exploratory variations between boys and girls in specific motor domains. Preliminary trends suggest that extracurricular structured PA might support adolescents with motor challenges, with future longitudinal studies required to further clarify these developmental pathways.

## Figures and Tables

**Table 1:** BMI categories for children and adolescents between ages 2 and 19 [53].

BMI Category	BMI Range
Underweight	Less than the 5th percentile
Healthy weight	5th percentile to less than the 85th percentile
Overweight	85th percentile to less than the 95th percentile
Obesity	95th percentile or greater
Severe Obesity	120% of the 95th percentile or greater, or 35 kg/m^2^ or greater

Note. According to CDC criteria, weight status was classified [53].

**Table 2:** Representation of differences in anthropometric characteristics among MABC-2 zones of motor competence (traffic light system).

MABC-2 Zones of Motor Competence (*N* = 742)							Statistics		
	No Motor Difficulties (Green Zone) (*n* = 704)		“At Risk” (Amber Zone) (*n* = 27)		Definite Motor Difficulties (Red Zone) (*n* = 11)				
Anthropometric Characteristics	Median	Mean Rank	Median	Mean Rank	Median	Mean Rank	Chi-Square (*χ*^2^) *df* = 2	*p*-Value	Eta Squared (*η*^2^)
Age (years)	13.90	370.64	13.75	334.63	14.50	517.32	5.90	0.052	0.005
Body Height (cm)	165.00	355.94	164.50	329.46	164.00	317.85	0.71	0.69	0.002
Body Weight (Kg)	55.00	354.01	56.50	364.02	63.00	435.55	1.60	0.45	0.001
BMI (Kg/m^2^)	20.22	351.42	21.03	388.85	23.55	481.30	4.64	0.09	0.004

Note. *χ*^2^ = Kruskal–Wallis test statistic; *df* = degrees of freedom; *p* = asymptotic significance level.

**Table 3:** Prevalence, 95% confidence intervals, and subscale distribution of MABC-2 (*N* = 742).

Movement ABC-2 Zones of Motor Difficulty “Traffic Light System”	Movement ABC-2 (*N* = 742)					
	Total MABC-2 Score			Manual Dexterity	Aiming/Catching	Static/ Dynamic Balance
	Total (*n*% [CI]) (*N* = 742)	Boys (*n*% [CI]) (*N* = 367)	Girls (*n*% [CI]) (*N* = 375)	Total (*n*% [CI]) (*N* = 742)		
No motor difficulties (green zone)	704 (94.9 [93.0–96.2])	345 (94 [91.1–96])	359 (95.7 [93.2–97.4])	670 (90.3 [88.0–92.2])	659 (88.8 [86.3–90.9])	711 (95.8 [94.1–97.0])
“At risk” (amber zone)	27 (3.6 [2.5–5.2])	16 (4.4 [2.7–7.0])	11 (2.9 [1.6–5.2])	52 (7.0 [5.4–9.1])	38 (5.1 [3.8–7.0])	19 (2.6 [1.6–4.0])
Definite motor Difficulties (red zone)	11 (1.5 [0.8–2.6])	6 (1.6 [0.8–3.5])	5 (1.4 [0.6–3.1])	20 (2.7 [1.8–4.1])	45 (6.1 [2.6–8.0])	12 (1.6 [0.9–2.8])

Note. *N* = 742; 367 boys and 375 girls; percentages (%) represent the prevalence within each column. Values in brackets [] indicate the 95% confidence interval (95% CI) calculated using the Wilson score method.

**Table 4:** Movement ABC-2 domain scores by gender across at-risk and definite motor difficulty zones.

**Movement ABC-2** **Zones of Motor Difficulty-** **“Traffic Light System”**	**Movement ABC-2 Motor Domains**	**Boys** **(*n* = 16)** ***M*** **(*SD*)**	**Girls** **(*n* = 11)** ***M*** **(*SD*)**	**Statistics**		**Effect** **Size**	**95% CI**
				***t*** **(*df* = 25)**	***p Value***	***Hedges’ g***	
“At risk” (amber zone) N = 27	Manual Dexterity	21.50 (5.75)	24.41 (3.99)	−1.45	0.15	−0.56	[−7.03, 1.22]
	Aiming/ Catching	15.69 (4.08)	12.27 (2.76)	2.41	0.02 *	0.81	[0.50, 6.32]
	Static/ Dynamic Balance	26.06 (6.60)	26.09 (3.59)	−0.01	0.99	−0.00	[−4.54, 4.48]
	Total MABC-2 score	63.25 (3.19)	62.77 (3.54)	0.36	0.71	0.14	[−2.21, 3.17]
**Movement ABC-2** **Zones of Motor Difficulty-** **“Traffic Light System”**	**Movement ABC-2 Motor Domains**	**Boys (*n* = 6)** ***M*** **(*SD*)**	**Girls** **(*n* = 5)** ***M*** **(*SD*)**	**Statistics**		**Effect** **Size**	**95% CI**
				***t*** **(*df* = 9)**	***p Value***	***Hedges’ g***	
Definite motor difficulties (red zone) N = 11	Manual Dexterity	11.50 (7.68)	18.80 (3.96)	−1.91	0.08	−1.09	[−15.94, 1.34]
	Aiming/ Catching	14.83 (3.43)	9.00 (2.91)	3.00	0.01 *	1.75	[1.43, 10.23]
	Static/ Dynamic Balance	14.17 (6.04)	18.40 (5.55)	−1.19	0.26	−0.70	[−12.22, 3.75]
	Total MABC-2 score	40.50 (6.86)	46.20 (7.12)	−1.34	0.21	−0.79	[−15.25, 3.85]

Note. *N* = 742; 367 boys and 375 girls; *M* = mean; *SD* = standard deviation; *t* = Student’s *t*-test statistic; *df* = degrees of freedom; *p* = statistical significance value; *d* = Hedges’ *g* effect size; MABC-2 = Movement Assessment Battery for Children—Second Edition; *p* < 0.05. * = Statistically significant at the 0.05 level.

**Table 5:** BMI classification by Movement ABC-2 motor difficulty zones with 95% CIs.

Movement ABC-2 Zones of Motor Difficulty—“Traffic Light System”	BMI Classification [53]					Total *N* = 742
	Underweight	Healthy Weight	Overweight	Obesity	Severe Obesity	
	*n* (% [95% CI])	*n* (% [95% CI])	*n* (% [95% CI])	*n* (% [95% CI])	*n* (% [95% CI])	*N* (100%)
No motor difficulties (green zone)	18 (2.5 [1.6–4.0])	552 (78.5 [75.2–81.3])	106 (15 [12.6–17.9])	28 (4 [2.8–5.7])	0 (0.0 [0.0–0.5])	704
“At risk” (amber zone)	1 (4.1 [0.7–18.3])	20 (75 [55.3–86.8])	4 (16.7 [5.9–32.5])	1 (4.2 [0.7–18.3])	0 (0.0 [0.0–25.9]	27
Definite motor difficulties (red zone)	0 (0.0 [0.0–25.9])	6 (54.6 [28.0–78.7])	3 (27.3 [9.7–56.6])	2 (18.1 [5.1–47.7])	0 (0.0 [0.0–25.9])	11

Note: *N* = 742. CI = confidence interval. Percentages represent the row-wise prevalence within each motor difficulty zone. Values in brackets [] indicate 95% confidence intervals calculated using the Wilson score method. No participants fell into the “Severe Obesity” category within the current sample (*n* = 0).

**Table 6:** Movement ABC-2 motor difficulty classifications by handedness with 95% CIs.

Handedness	Movement ABC-2 Zones of Motor Difficulty “Traffic Light System”			
	No Motor Difficulties (Green Zone)	“At Risk” (Amber Zone)	Definite Motor Difficulties (Red Zone)	Total (100%) *N* = 742
	*n* (% [95% CI])	*n* (% [95% CI])	*n* (% [95% CI])	*N*
Right-handed	638 (95.1% [93.2–96.5])	22 (3.3% [2.2–4.9])	11 (1.6% [0.9–2.9])	671
Left-handed	66 (93.0% [84.6–97.0])	5 (7% [3.0–15.4])	0 (0.0% [0.0–5.1])	71

Note: *N* = 742. CI = confidence interval. Percentages represent the row-wise prevalence within each handedness group. Values in brackets [] indicate 95% confidence intervals calculated using the Wilson score method.

**Table 7:** Movement ABC-2 motor difficulty classifications by secondary school grades with 95% CIs.

Secondary School Grades	Movement ABC-2 Zones of Motor Difficulty “Traffic Light System”			
	No Motor Difficulties (Green Zone)	“At Risk” (Amber Zone)	Definite Motor Difficulties (Red Zone)	Total (100%) *N* = 742
	*n* (% [95% CI])	*n* (% [95% CI])	*n* (% [95% CI])	*N*
1st Grade (*M_age_* ± *SD*) = (12.64 ± 0.46)	44 (93.6 [82.8–97.8])	2 (4.3 [1.2–14.2])	1 (2.1 [0.4–11.1])	47
2nd Grade (*M_age_* ± *SD*) = (13.65 ± 0.42)	392 (94.9 [92.4–96.7])	18 (4.4 [2.8–6.8])	3 (0.7 [0.2–2.1])	413
3rd Grade (*M_age_* ± *SD*) = (14.70 ± 0.44)	268 (95.0 [91.8–97.0])	7 (2.5 [1.2–5.0])	7 (2.5 [1.2–5.0])	282

Note. *N* = 742; (*M_age_* ± *SD*): means and standard deviations for age among grades, CI = confidence interval. Percentages represent the row-wise prevalence within each school grade level. Values in brackets [] indicate 95% confidence intervals calculated using the Wilson score method.

**Table 8:** Extracurricular organized PA participation by Movement ABC-2 Motor difficulty zones with 95% CIs.

Movement ABC-2 Zones of Motor Difficulty—“Traffic Light System”	Extracurricular Organized PA Participation		
	PA Participation	No PA Participation	Total (100%) *N* = 742
	*n* (% [95% CI])	*n* (% [95% CI])	*N*
No motor difficulties (green zone)	279 (39.6 [36.1–43.3])	425 (60.4 [56.7–63.9])	704
“At risk” (amber zone)	9 (33.3 [18.6–52.2])	18 (66.7 [47.8–81.4])	27
Definite motor difficulties (red zone)	4 (36.4 [15.2–64.6])	7 (63.6 [35.4–84.8])	11

Note: *N* = 742. PA = physical activity; CI = confidence interval. Percentages represent row-wise prevalence within each motor difficulty zone. Values in brackets [] indicate 95% confidence intervals calculated using the Wilson score method.

**Table 9:** Movement ABC-2 domain scores by extracurricular organized PA participation across at-risk and definite motor difficulty zones.

**Movement ABC-2** **Zones of Motor** **Difficulty—“Traffic Light System”**	**Movement ABC-2 Motor Domains**	**Extracurricular Organized PA Participation**		**Statistics**		**Effect** **Size**	**95% CI**
		**Yes (*n* = 9)** ***M*** **(*SD*)**	**No (*n* = 18)** ***M*** **(*SD*)**	***t*** **(*df* = 25)**	***p Value***	***Hedges’ g***	
“At risk” (amber zone) *N* = 27	Manual Dexterity	19.56 (4.47)	24.25 (4.95)	−2.39	0.02 *	−0.95	[−8.73, −0.65]
	Aiming/Catching	14.89 (3.44)	14.00 (4.21)	0.54	0.59	0.22	[−2.46, 4.23]
	Static/Dynamic Balance	30.11 (4.91)	24.06 (4.67)	3.12	0.00 *	1.23	[2.06, 10.05]
	Total MABC-2 score	64.56 (3.24)	62.31 (3.12)	1.74	0.09	0.69	[−0.41, 4.9]
**Movement ABC-2** **Zones of Motor** **Difficulty—“Traffic Light System”**	**Movement ABC-2 Motor Domains**	**Yes (*n* = 4)** ***M*** **(*SD*)**	**No (*n* = 7)** ***M*** **(*SD*)**	**Statistics**		**Effect** **Size**	**95% CI**
				***t*** ***(df = 9)***	***p Value***	***Hedges’ g***	
Definite Motor difficulties (red zone) *N* = 11	Manual Dexterity	12.00 (7.74)	16.43 (6.75)	−0.99	0.34	−0.57	[−14.49, 5.63]
	Aiming/Catching	15.50 (3.31)	10.29 (3.72)	2.31	0.04 *	1.33	[0.11, 10.31]
	Static/Dynamic Balance	16.00 (5.59)	16.14 (6.59)	−0.03	0.97	−0.02	[−9.04, 8.76]
	Total MABC-2 score	43.50 (5.74)	42.86 (8.43)	0.13	0.89	0.07	[−10.19, 11.48]

Note. *N* = 742; *M* = mean; *SD* = standard deviation; *t* = Student’s *t*-test statistic; *df* = degrees of freedom; *p* = statistical significance value; *d* = Hedges’ g effect size; MABC-2 = Movement Assessment Battery for Children—Second Edition; *p* < 0.05. * = Statistically significant at the 0.05 level.

**Table 10:** Movement ABC-2 motor difficulty classifications by type of school with 95% CIs.

Type of School	Movement ABC-2 Zones of Motor Difficulty “Traffic Light System”			
	No Motor Difficulties (Green Zone)	“At Risk” (Amber Zone)	Definite Motor Difficulties (Red Zone)	Total (100%) *N* = 742
	*n* (% [95% CI])	*n* (% [95% CI])	*n* (% [95% CI])	*N*
Standard public school	533 (92.5 [90.1–94.4])	33 (5.8 [4.1–7.9])	10 (1.7 [0.9–3.2])	576
Public music school	90 (100 [95.9–100.0])	0 (0.0%) [0.0–4.1])	0 (0.0 [0.0–4.1])	90
Standard private school	70 (92.1 [83.8–96.3])	4 (5.3 [2.1–12.8])	2 (2.6 [0.7–9.1])	76

Note: *N* = 742. CI = confidence interval. Percentages represent row-wise prevalence within each school type. Values in brackets [] indicate 95% confidence intervals, calculated using the Wilson score method.

## Data Availability

The data presented in this study are available on request from the corresponding author due to privacy reasons.

## Abbreviations

The following abbreviations are used in this manuscript:

MABC-2	Movement Assessment Battery for Children—2nd Edition
DCD	Developmental Coordination Disorder
DSM-5	Diagnostic and Statistical Manual of Mental Disorders, Fifth Edition
PA	Physical Activity
PE	Physical Education
BMI	Body Mass Index
SS	Standard Score
CSS	Component Standard Score
